# Odontogenic Ameloblast-associated Protein (ODAM) Mediates Junctional Epithelium Attachment to Teeth via Integrin-ODAM-Rho Guanine Nucleotide Exchange Factor 5 (ARHGEF5)-RhoA Signaling*

**DOI:** 10.1074/jbc.M115.648022

**Published:** 2015-04-24

**Authors:** Hye-Kyung Lee, Suk Ji, Su-Jin Park, Han-Wool Choung, Youngnim Choi, Hyo-Jung Lee, Shin-Young Park, Joo-Cheol Park

**Affiliations:** From the ‡ Departments of Oral Histology/Developmental Biology and; ¶ Immunology and Molecular Microbiology, School of Dentistry and Dental Research Institute, Seoul National University, 101 Daehagro, Chongro-gu, Seoul 110-744, Korea,; the § Department of Periodontology, Anam Hospital, Korea University, 73 Inchonro, Anam-dong, Seongbuk-gu, Seoul 136-713, Korea, and; the ‖ Department of Periodontology, Section of Dentistry, Seoul National University Bundang Hospital, 173-82 Gumiro, Seongnam-si, Gyeonggi-do 463-707, Korea

**Keywords:** ODAM, tooth, junctional epithelium, Rho signaling, periodontitis

## Abstract

Adhesion of the junctional epithelium (JE) to the tooth surface is crucial for maintaining periodontal health. Although odontogenic ameloblast-associated protein (ODAM) is expressed in the JE, its molecular functions remain unknown. We investigated ODAM function during JE development and regeneration and its functional significance in the initiation and progression of periodontitis and peri-implantitis. ODAM was expressed in the normal JE of healthy teeth but absent in the pathologic pocket epithelium of diseased periodontium. In periodontitis and peri-implantitis, ODAM was extruded from the JE following onset with JE attachment loss and detected in gingival crevicular fluid. ODAM induced RhoA activity and the expression of downstream factors, including ROCK (Rho-associated kinase), by interacting with Rho guanine nucleotide exchange factor 5 (ARHGEF5). ODAM-mediated RhoA signaling resulted in actin filament rearrangement. Reduced ODAM and RhoA expression in *integrin* β*_3_*- and β*_6_*-knockout mice revealed that cytoskeleton reorganization in the JE occurred via integrin-ODAM-ARHGEF5-RhoA signaling. Fibronectin and laminin activated RhoA signaling via the integrin-ODAM pathway. Finally, ODAM was re-expressed with RhoA in regenerating JE after gingivectomy *in vivo*. These results suggest that ODAM expression in the JE reflects a healthy periodontium and that JE adhesion to the tooth surface is regulated via fibronectin/laminin-integrin-ODAM-ARHGEF5-RhoA signaling. We also propose that ODAM could be used as a biomarker of periodontitis and peri-implantitis.

## Teaser

**Conclusion:** ODAM mediates JE attachment to healthy teeth.

**Significance:** We investigate ODAM function during JE development and regeneration and its functional significance in the initiation and progression of periodontal disease.

## Introduction

The junctional epithelium (JE)^2^ is a specialized epithelial structure that attaches the gingival soft tissue to the tooth surface (1). In periodontal disease, oral microbes and the host response induce the JE to migrate apically and invade the gingival connective tissue during its transformation to pocket epithelium. Inflammation around the pocket epithelium leads to the resorption of alveolar bone around the tooth and, therefore, to the loss of the periodontal ligament attachment, which is normally responsible for suspending the tooth within the bone (2). Therefore, the JE represents the first line of defense against prevalent periodontal diseases (3, 4). Breakdown of the JE attachment to the tooth surface in the development of periodontal disease has significant consequences for oral health.

The JE is derived from reduced enamel epithelium. After the tip of the tooth approaches the oral mucosa during tooth eruption, the reduced enamel epithelium and the oral epithelium meet, fuse, and form the dentogingival junction (5). However, reduced enamel epithelium is not essential for JE regeneration because it is completely restored from the adjacent sulcular or oral epithelium after pocket instrumentation or surgery. Newly regenerated JE exhibits the same structural and functional features as the original JE (6). However, the molecular mechanisms responsible for inducing the formation of the JE during regeneration remain unclear.

The odontogenic ameloblast-associated protein (ODAM) has been implicated in diverse activities, such as ameloblast differentiation, enamel maturation, and tumor growth (7–10). ODAM is expressed during the developmental continuum from maturation stage ameloblasts to normal JE but is reduced after JE damage (6, 11–14). ODAM is re-expressed in regenerated JE after orthodontic tooth movement and surgical excision (11, 12). However, the functional role of ODAM in regenerating JE has not yet been established.

Epithelial integrins also participate in the regulation of periodontal inflammation (15). Integrins are cell adhesion receptors that link the extracellular matrix to the cellular cytoskeleton, including fibronectin and collagens (16). Integrin α_v_β_3_ is crucial for bone-resorbing function in periodontal disease (17). Integrin α_v_β_6_ is constitutively expressed in human and murine JE, and *integrin* β*_6_*^−/−^ mice develop all of the classic hallmarks of chronic periodontal disease as the initial signs of periodontal disease (18).

During amelogenesis, ameloblasts undergo dramatic cytoskeletal changes, and RhoA protein levels are up-regulated (19). Rho guanine nucleotide exchange factor 5 (ARHGEF5/TIM) belongs to the Rho-GEF family and has GDP-GTP exchange activity for RhoA (20). Arhgef5 can strongly activate RhoA and RhoB and stimulate Arhgef5-mediated activation of RhoA in dendritic cell chemotaxis (21). However, although RhoA and ARHGEF5 are expressed in ameloblasts and JE, the RhoA-ARHGEF5 pathway in amelogenesis and JE formation remains unclear.

The objectives of this study were to investigate the mechanism of JE attachment to the tooth surface for the formation of an epithelial barrier against periodontal pathogens in healthy and inflamed periodontal tissues. We also identified epithelial attachment loss using objective measures such as biomarkers in the gingival crevicular fluid (GCF) after destruction and apical migration of JE. We tested the hypothesis that certain extracellular matrix molecules induce ODAM expression in JE via integrin receptors and that ODAM subsequently triggers cytoskeletal changes of the JE via ARHGEF5-RhoA signaling during dentogingival junction development and regeneration. In addition, we evaluated ODAM protein levels in GCF from periodontitis and peri-implantitis patients for early diagnosis and progress monitoring of periodontal disease.

## Experimental Procedures

### 

#### 

##### Reagents and Antibodies

The anti-ODAM antibody was generated in rabbits by immunization with ODAM peptides (22). Anti-RhoA, F-actin, GAPDH, HA, ROCK, His, lamin B, integrin β_1_, integrin β_3_, integrin β_6_, HRP-conjugated goat anti-mouse, HRP-conjugated goat anti-rabbit-IgG, and HRP-conjugated rabbit anti-goat-IgG antibodies were purchased from Santa Cruz Biotechnology (Santa Cruz, CA). Anti-RhoA, E-cadherin, p-myosin, p-paxillin, and paxillin antibodies as well as integrin β_1_ and integrin β_6_ siRNA were obtained from Cell Signaling Technology (Beverly, MA). The anti-GTP-RhoA antibody was purchased from BIOSOURCE. Anti-Arhgef5 was obtained from Proteintech Group (Chicago, IL). Anti-FLAG and transglutaminase 2 (TG2) antibodies, fibronectin, laminin, collagen, and *Porphyromonas gingivalis* LPS were from Sigma-Aldrich (St. Louis, MO). The Alexa Fluor 488 phalloidin (rhodamine-phalloidin) antibody was obtained from Invitrogen. Anti-FITC or Cy3-conjugated anti-mouse, rabbit, or goat IgG antibodies were purchased from Life Technologies. Y-27632 for ROCK inhibition was obtained from Tocris Cookson (Avonmouth, UK).

##### Plasmids, Cloning, and Recombinant ODAM

cDNAs of full-length ODAM or its deletion mutants, siRNA targeting ODAM, and pGL3-Dspp vectors were constructed and verified as described previously (22). His-fused ODAM proteins were extracted and purified as described previously (7). The GFP-tagged RhoAQ63L (constitutively active RhoA) construct was provided by Dr. Hyun-Man Kim (Seoul National University, Seoul, Korea). Full-length FLAG-tagged Arhgef5, ΔPH (amino acids 1341–1488), and Arhgef5 ΔDH (amino acids 1064–1340) were provided by Dr. Masato Okada (Osaka University, Osaka, Japan). The pOTB7-Arhgef5 construct was purchased from the Korea Human Gene Bank. FLAG-tagged Arhgef5 ΔSH and SH (amino acids 1489–1581) were subcloned into FLAG-tagged pcDNA3 (Invitrogen).

##### Experimental Periodontitis

Experimental periodontitis in mice was induced by *P. gingivalis* (PG) inoculation and dextran sulfate sodium (DSS) treatment. Mice were randomly divided into three groups: sham, DSS, and PG. The DSS group received daily application of 5% DSS (MP Biomedicals, Irvine, CA). The PG group received oral inoculation of 10^9^ cells of PG cells in 100 μl of 2% carboxymethylcellulose on days 4, 6, and 8. The sham group received vehicles instead of DSS and PG. All mice were euthanized on day 50.

##### Tissue Preparation and Immunohistochemistry

All animal experiments were performed according to the Dental Research Institute guidelines of Seoul National University. Teeth blocks from WT and *integrin* β*_3_*^−/−^ mice were provided by Dr. Toshiyuki Yoshida and Teruo Okano (Tokyo Women's Medical University, Tokyo, Japan). Extracted human teeth and associated gingival tissue were obtained from Seoul National University Dental Hospital. These studies were approved by the Institutional Review Board for Human Subjects of the Seoul National University (IRB no. S-D20140007). Rat and mouse teeth were decalcified in 10% EDTA (pH 7.4), embedded in paraffin, and processed for immunohistochemistry. Sections were incubated overnight at 4 °C with primary antibodies (dilutions of 1:100–1:200). Secondary anti-rabbit or anti-mouse IgG antibodies were added to the sections for 30 min at room temperature, followed by reaction with the avidin-biotin-peroxidase complex (Vector Laboratories, Burlingame, CA). Signals were converted using a diaminobenzidine kit (Vector Laboratories). Nuclei were stained with hematoxylin.

##### Gene Expression Profiling

Gene expression profile data (GSE2429) were obtained from the National Center for Biotechnology Information Gene Expression Omnibus (NCBI GEO) database (accession number GSE10526 to PG SerB mutant infection effect on immortalized gingival epithelial cells, GSE4250 to hereditary gingival fibromatosis, and GSE2255 to integrin β_6_ deficiency model of emphysema).

##### Study Subjects and Clinical Examinations

After informed consent, 14 unrelated, systemically healthy adults were included in the study. This study protocol was approved by the Institutional Review Board for Human Subjects of the Korea University Anam Hospital (IRB no. ED13162). Periodontal examination included the assessment of plaque score, probing pocket depth, loss of attachment, and bleeding on probing. For peri-implantitis evaluation, two patients with peri-implantitis were included in the study, and two healthy implants served as control. This protocol was approved by the Institutional Review Board for Human Subjects of Seoul National University Bundang Hospital (IRB no. B-1410-271-003).

##### GCF Collection and ELISA

Samples were obtained from teeth of one quadrant on the jaw that contained the teeth showing the deepest probing depth and the contralateral quadrant of the opposite jaw. Therefore, a total of 222 samples were collected from 12–16 teeth of each subject. Each tooth site was gently dried for 10 s with compressed air and isolated from saliva with a cotton roll. GCF samples were obtained from four sites of one tooth using absorbent paper strips (Oraflow Inc., Plainview, NY). Paper strips were placed in a single labeled tube containing 100 μl of PBS. The total levels of ODAM in GCF samples were assayed using an ODAM ELISA kit according to the instructions of the manufacturer (Cusabio Biotech, Wuhan, China). Associations between probing depths and ODAM concentrations in GCF were analyzed using a Kruskal-Wallis test and SPSS.

##### Cell Culture and Transient Transfection

Ameloblast lineage cells (ALCs) were cultured on collagen-coated dishes in minimum essential medium supplemented with 5% FBS, 10 ng/ml recombinant human EGF (Sigma-Aldrich), and an antibiotic/antimycotic agent (Invitrogen) in 5% CO_2_ at 37 °C. HAT7 cells, a dental epithelial cell line originating from a cervical loop epithelium of a rat incisor (a gift from Dr. Harada, Department of Oral Anatomy II, Iwate Medical College School of Dentistry, Morioka, Japan), were grown and maintained in DMEM/F12 (Gibco). RAW264.7 cells, a macrophage-like cell line derived from BALB/c mice, were grown and maintained in DMEM. To induce differentiation, 80–90% confluent cells were cultured in minimum Eagle's medium supplemented with 5% FBS, ascorbic acid (50 μg/ml), and β-glycerophosphate (10 mm) for up to 2 weeks. ALC or HAT7 cells were seeded in culture plates. Cells were transiently transfected with reporter constructs using Metafectene PRO reagent (Biontex, Planegg, Martinsried, Germany). In addition, cells were transiently transfected with siRNA (Santa Cruz Biotechnology) using Lipofectamine RNAi MAX reagent (Invitrogen).

##### Immunoprecipitation Assay and His Pulldown Assay

Cell lysates were prepared by adding 1 ml of radioimmune precipitation assay buffer (50 mm Tris-Cl (pH 7.5), 150 mm NaCl, 1% Nonidet P-40, 1 mm EDTA, 1 mm PMSF, 1 mm Na_3_VO_4_, and 1 mm NaF) supplemented with protease inhibitors (Roche Molecular Biochemicals, Mannheim, Germany). Lysates were incubated at 4 °C for 2 h with a 1:200 dilution of the indicated antibody. After incubation for 2 h at 4 °C with A/G-agarose beads (Santa Cruz Biotechnology), the beads were washed three times with radioimmune precipitation assay buffer. Immune complexes were released from the beads by boiling. Following electrophoresis on 10% SDS-polyacrylamide gels, immunoprecipitates were analyzed by Western blot using the indicated antibodies.

For His pulldown assays, 24 h after transfection, cells were lysed in radioimmune precipitation assay buffer. Lysates were incubated for 1 h at 30 °C with His-ODAM C-terminal protein, followed by incubation for 2 h at 4 °C with a 1:200 dilution of the anti-His antibody. After incubation for 2 h at 4 °C with A/G-agarose beads (Santa Cruz Biotechnology), beads were washed three times with radioimmune precipitation assay buffer, and immune complexes were released from the beads by boiling. Following electrophoresis on 10% SDS-polyacrylamide gels, immunoprecipitates were analyzed by Western blot using the indicated antibodies.

##### Preparation of Cytoplasmic and Nuclear Protein Extracts

Cells were collected by centrifugation. Cells were lysed in ice-cold hypotonic lysis buffer (10 mm HEPES (pH 7.9), 10 mm KCl, and 0.1% Nonidet P-40) supplemented with protease inhibitors (Roche). Nuclear and cytoplasmic fractions were separated by centrifugation. The membrane pellet was resuspended in ice-cold hypertonic lysis buffer (10 mm HEPES (pH 7.9), 150 mm NaCl, 1% Nonidet P-40, 0.25% sodium deoxycholate, and 10% glycerol). The soluble fraction was isolated by centrifugation.

##### Western Blot Analysis

Proteins (30 μg) from the cells were separated by 10% SDS-PAGE and transferred to nitrocellulose membranes. Membranes were blocked for 1 h with 5% nonfat dry milk in PBS-T buffer (PBS containing 0.1% Tween 20), and incubated overnight at 4 °C with the primary antibody diluted in PBS-T buffer (1:1000). After washing, membranes were incubated for 1 h with secondary antibodies. Labeled protein bands were detected using an enhanced chemiluminescence system (Dogen, Cambridge, MA).

##### Fluorescence Microscopy

Cells in Laboratory-Tek chamber slides (Nunc, Rochester, NY) were washed with PBS, fixed with 4% paraformaldehyde in PBS, and permeabilized in PBS containing 0.5% Triton X-100. After washing and blocking, Cells were incubated for 1 h with primary (1:200) and Alexa Fluor 488 phalloidin antibodies in blocking buffer (PBS and 1% bovine serum albumin), followed by the addition of anti-FITC or Cy3-conjugated anti-mouse, rabbit, or goat IgG antibodies (1:200). After washing, cells were visualized using fluorescence microscopy (AX70, Olympus Optical Co, Tokyo, Japan). Chromosomal DNA in the nucleus was stained using DAPI.

##### RhoA Activity Assay

GTP-loaded RhoA levels were determined using the RhoA G-LISA Activation Assay Kit (Cytoskeleton, Denver, CO) according to the instructions of the manufacturer. Equal amounts of proteins from each experimental group were used in G-LISA RhoA activation assays to obtain values for RhoA activity per cell.

##### Cell Adhesion Assay

ALC cells were seeded on slides coated with recombinant ODAM protein (rODAM) or collagen and incubated for 4 h. Cells were fixed with 4% paraformaldehyde for 30 min and stained with crystal violet for 10 min, and, finally, the optical density at 595 nm was measured.

##### Periodontal Challenge Procedures

Thirty healthy upper first molars from 24 8-week-old Sprague-Dawley male rats were used for gingivectomy. Surgical areas were cleaned with 0.5% chlorhexidine. Removal of the gingiva and the JE along the maxillary molars (gingivectomy) was accomplished by scraping or ligature of the tooth surface and extended 2 mm along the palate.

##### Statistical Analyses

All quantitative data are presented as the mean ± S.D. Statistical differences were analyzed using Student's *t* tests (*, *p* < 0.05).

## Results

### 

#### 

##### ODAM Expression Was Reduced after Inflammation or Chemical Damage in JE

**FIGURE 1.**
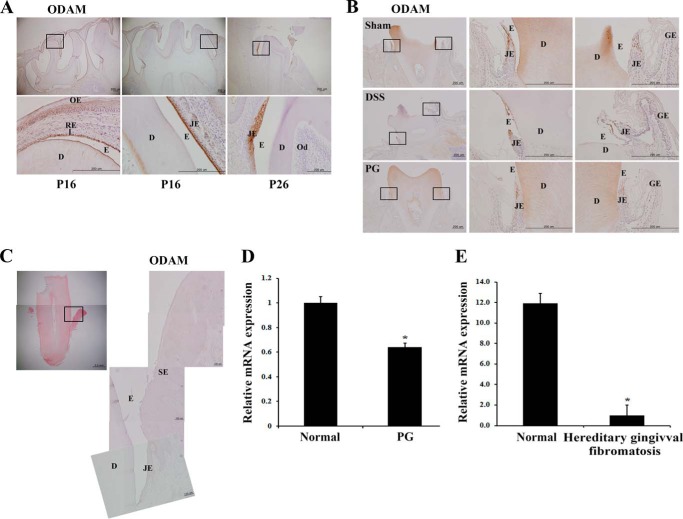
**ODAM was expressed in normal JE but reduced after inflammation or damage.** *A*, immunohistochemistry indicates that ODAM was expressed in reduced enamel epithelium (*left panels*), maturation-stage ameloblasts (*central panels*), and JE (*right panels*) during rat tooth development on postnatal days 16 (*P16*) and P26. *Scale bars* = 200 μm. *OE*, oral epithelium; *RE*, reduced epithelium; D, dentin; *E*, enamel; *Od*, odontoblast. *B*, ODAM expression was reduced after inflammation by DSS treatment and PG inoculation in JE of 6-week-old mice (3 mice/treatment group). *Scale bar* = 200 μm. *GE*, gingival epithelium. *C*, gingival sections from periodontitis patients did not express ODAM (*n* = 4). *Scale bars* = 100 μm. *SE*, sulcular epithelium. *D*, the expression of *ODAM* mRNA was analyzed from gene expression dataset GSE10526 deposited in the GEO (*n* = 4). *E*, expression of *ODAM* mRNA was analyzed from gene expression dataset GSE4250 deposited in the GEO (*n* = 2). *, values significantly different from control (*p* < 0.05).

ODAM was expressed in differentiating ameloblasts as well as in normal and regenerating JE (6, 23). First, we investigated ODAM protein expression during amelogenesis and JE formation by immunohistochemistry. ODAM was clearly observed in reduced enamel epithelium, maturation-stage ameloblasts, and JE during rat tooth development (Fig. 1*A*). ODAM expression was reduced in JE after damage by chemical drugs, DSS, and PG compared with the sham group (Fig. 1*B*). To investigate whether periodontitis affects ODAM expression in human JE, we immunohistochemically evaluated ODAM protein expression in a human tooth extracted because of severe periodontitis. In the extracted tooth, JE transformed to the invasive pocket epithelium. In the pocket epithelium, ODAM was no longer detected (Fig. 1*C*). ODAM expression decreased significantly in damaged gingival epithelial cells modulated with the oral pathogenic PG compared with normal epithelial cells (Fig. 1*D*). To confirm the alteration of ODAM expression after inflammation in JE, we analyzed microarray data from the NCBI GEO dataset. Hereditary gingival fibromatosis associated with aggressive periodontitis typically results in severe, rapid destruction of the tooth-supporting apparatus (24). GEO data showed that ODAM expression decreased significantly in gingival tissues with hereditary gingival fibromatosis compared with those of normal patients (Fig. 1*E*). These results suggest that ODAM was expressed in normal JE of healthy tooth but decreased after inflammation or chemical damage and, consequently, disappeared in the pathologic pocket epithelium of diseased periodontium.

##### ODAM Was Detected in GCF from Periodontitis and Peri-implantitis Patients

**FIGURE 2.**
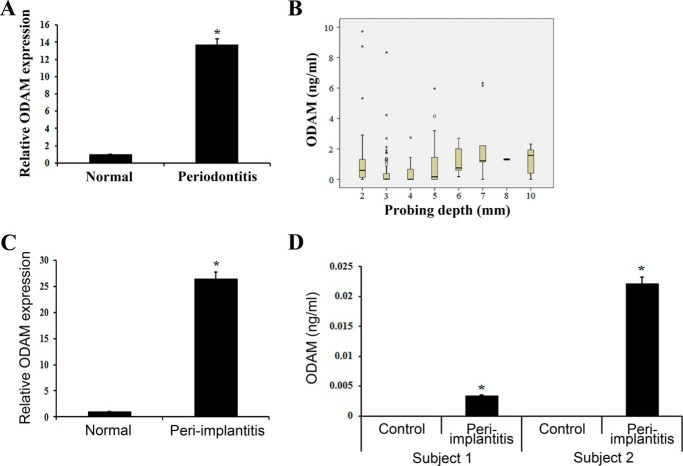
**ODAM was detected in GCF from periodontitis and peri-implantitis patients.** *A*, ODAM protein levels in GCF from healthy teeth (normal) and periodontitis patients were measured by ELISA. Summary results from 10 patients are shown. *B*, the association between probing depths and ODAM concentration in the GCF by ELISA was analyzed using a Kruskal-Wallis test (*n* = 4). *C*, ODAM protein in GCF from healthy teeth (normal) and peri-implantitis patients was measured by ELISA (*n* = 2/group). *D*, ODAM protein in GCF from control and peri-implantitis patients was measured by ELISA. Healthy implants served as controls (*n* = 2). Data are mean ± S.D. of triplicate experiments. *, *p* < 0.05 compared with the control.

ODAM protein was detected in sera from late-stage breast cancer patients (25). We found that ODAM was expressed in normal JE. However, its expression disappeared in pathologic pocket epithelium from periodontitis patients. On the basis of these findings, we investigated the expression of ODAM in GCF from periodontitis and peri-implantitis patients by ELISA. As expected, the level of ODAM protein was increased significantly in GCF from periodontitis patients compared with healthy teeth without inflammation (Fig. 2*A*). Furthermore, the level of ODAM protein in GCF correlated with the probing depth in periodontitis patients (Fig. 2*B*). Similar to periodontitis, the ODAM protein level was also increased significantly in GCF from peri-implantitis patients compared with healthy teeth (Fig. 2*C*) and healthy implants (Fig. 2*D*). These results demonstrate that ODAM expression in JE reflects a healthy periodontium. However, after JE attachment loss caused by periodontitis or peri-implantitis, ODAM is extruded from JE and detected in GCF.

##### ODAM Interacted with ARHGEF5 in Ameloblasts

**FIGURE 3.**
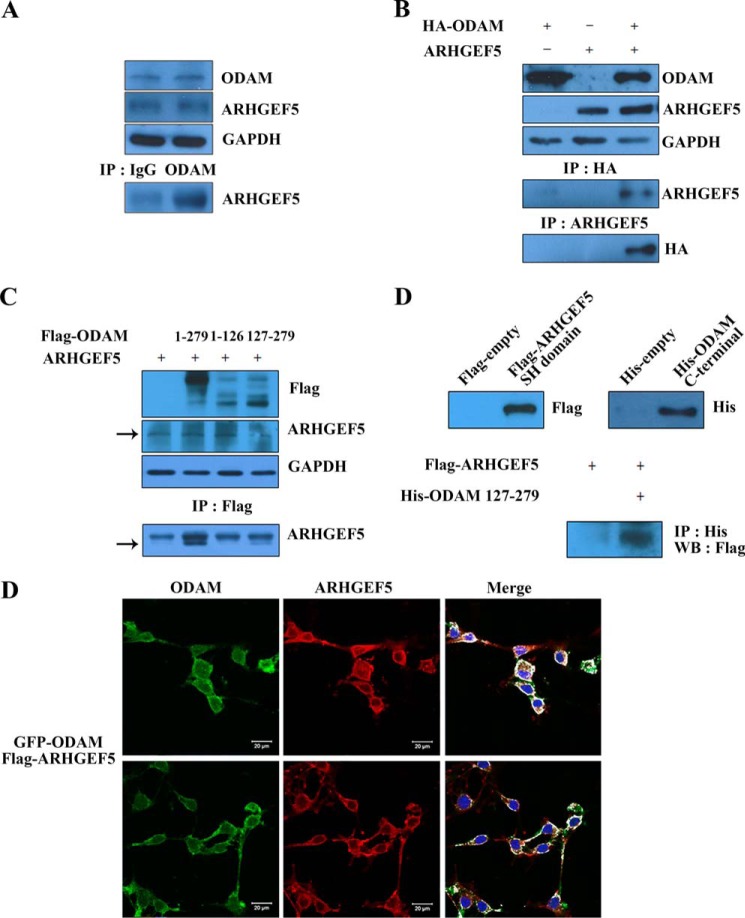
**ODAM interacted with ARHGEF5 in ameloblasts.** *A*, IP was performed using anti-ODAM antibody in ALCs. Precipitated proteins were visualized by Western blotting using anti-ARHGEF5 antibody. *B*, ALCs were cotransfected with HA-*ODAM* and *ARHGEF5* constructs. IP was performed using anti-HA or ARHGEF5 antibodies. Precipitated proteins were visualized by Western blotting using anti-ARHGEF5 or HA antibodies. *C*, mapping of the ODAM domain required for interaction with ARHGEF5. FLAG-*ODAM* mutants were expressed in ALCs transfected with *ARHGEF5*. The interaction was evaluated by IP using the anti-FLAG antibody, followed by Western blotting using anti-ARHGEF5 antibody. *D*, ALCs were transfected with the FLAG-*ARHGEF5* mutant containing only the SH domain (amino acids 1489–1581). His pulldown assays were performed with cells expressing the *ARHGEF5* SH domain. The ARHGEF5 interaction was determined by pulldown using the His-ODAM C-terminal mutant. Interactions were detected by Western blotting (*WB*) using an antibody specific for the FLAG tag expressed by the *ARHGEF5* mutant. *E*, GFP-tagged *ODAM* and FLAG-tagged *ARHGEF5* constructs were transfected into ALCs. Exogenous ARHGEF5 was immunostained using the anti-FLAG antibody, and GFP-ODAM was detected by immunofluorescence. *Scale bars* = 20 μm.

In our previous study, ARHGEF5 was identified as an ODAM-interacting protein by protoarray analysis (22). In immunoprecipitation (IP) assay, ODAM also showed endogenous interaction with ARHGEF5 in ALCs (Fig. 3*A*). To confirm whether ODAM could interact with ARHGEF5, ALCs were cotransfected with *ARHGEF5*and HA-tagged *ODAM* constructs for IP assay. The results demonstrated the interaction of ODAM with ARHGEF5 (Fig. 3*B*). IP with the FLAG antibody followed by blotting with the ARHEGF5 antibody indicated that amino acids 127–279 of ODAM affected the interaction between ODAM and ARHGEF5 (Fig. 3*C*). Pulldown assays also showed a direct interaction between these two proteins (Fig. 3*D*). Immunofluorescence microscopy revealed that the majority of GFP-tagged ODAM and FLAG-tagged ARHGEF5 proteins colocalized to the periphery of ALCs (Fig. 3*E*). Overall, these data suggest that the interaction between the C terminus of ODAM and the SH domain of ARHGEF5 occurs in the cell periphery of ameloblasts.

##### ODAM Mediated RhoA Signaling in Ameloblasts and JE

**FIGURE 4.**
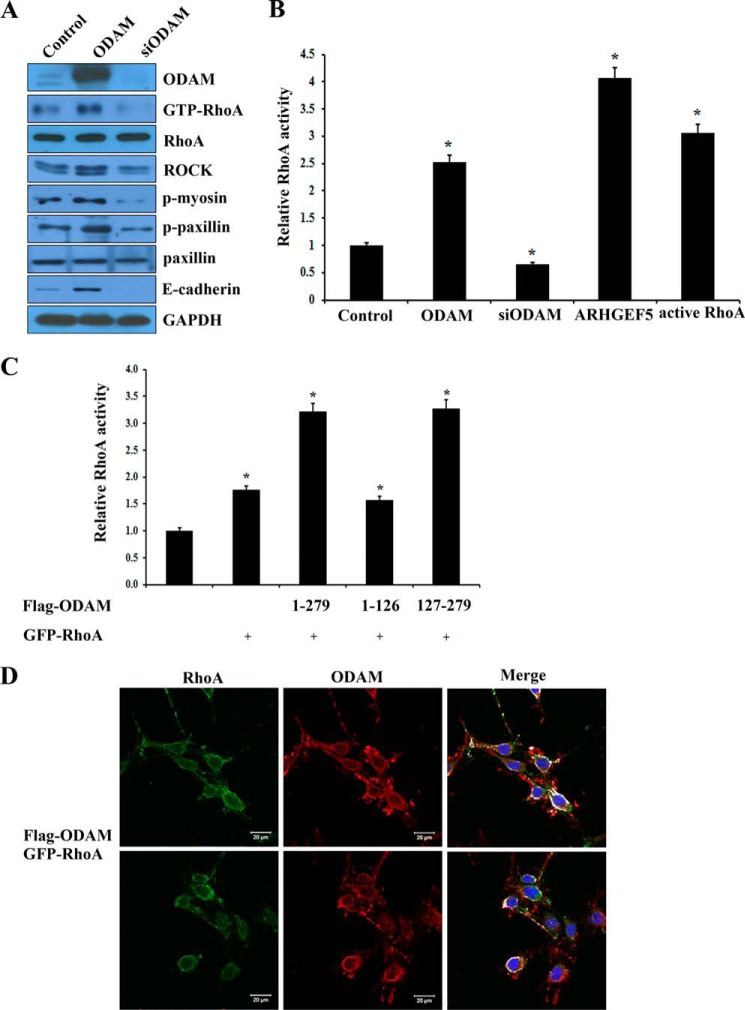
**ODAM induced RhoA signaling pathway in ameloblasts.** *A*, ALCs were transfected with *ODAM* or *ODAM* siRNA constructs. RhoA signaling component expression was analyzed by Western blot. *B*, ALCs were transfected with *ODAM*, *ODAM* siRNA, *ARHGEF5*, or active *RhoA* constructs. Equal amounts of cell lysates were used for G-LISA RhoA activation assays. *C*, mapping the ODAM domain required for RhoA activation with ARHGEF5. FLAG-*ODAM* mutants were expressed in ALCs transfected with the ARHGEF5 construct. RhoA activity was determined by G-LISA RhoA activation assays. Data are mean ± S.D. of triplicate experiments. *, *p* < 0.05 compared with the control. *D*, FLAG-tagged *ODAM* and GFP-tagged *RhoA* constructs were transfected into ALCs. Exogenous ODAM was immunostained using anti-FLAG antibody, and GFP-RhoA was detected by immunofluorescence. Nuclei were stained with DAPI. *Scale bars* = 20 μm.

GEFs-activated RhoA regulates downstream effectors, including ROCK and myosin (26). To investigate the effects of ODAM on RhoA signaling during amelogenesis, we examined the expression levels of RhoA downstream factors, including ROCK, p-myosin, p-paxillin, and E-cadherin. *ODAM* overexpression increased the phosphorylation activity of RhoA, myosin, and paxillin as well as the expression of ROCK and E-cadherin, whereas siRNA-mediated *ODAM* inactivation decreased their activity and expression (Fig. 4*A*). However, the total expression of RhoA and paxillin were unaffected by *ODAM* overexpression or inactivation. RhoA signaling was robust in *ODAM*-, *ARHGEF5*-, and active *RhoA*-expressing ALCs but inhibited after siRNA-mediated *ODAM* inactivation (Fig. 4*B*). To map the ODAM functional domain required for RhoA activation with *ARHGEF5*, we performed a RhoA activity assay using *ODAM* deletion constructs. RhoA activation demonstrated that deletion of the C-terminal region of *ODAM* (amino acids 127–279) affected RhoA activation with *ARHGEF5* (Fig. 4*C*). This result suggests that the C-terminal domain containing the amino acid 127–279 region of *ODAM* is necessary for activation of RhoA signaling with *ARHGEF5*. Confocal microscopy showed that FLAG-tagged ODAM and GFP-tagged RhoA proteins primarily colocalized to the cell periphery of ALCs (Fig. 4*D*). These data suggest that ARHGEF5-ODAM mediates the activation of RhoA signaling in ameloblasts and JE.

##### ODAM-mediated RhoA Signaling Resulted in Cytoskeleton Reorganization in Ameloblasts

**FIGURE 5.**
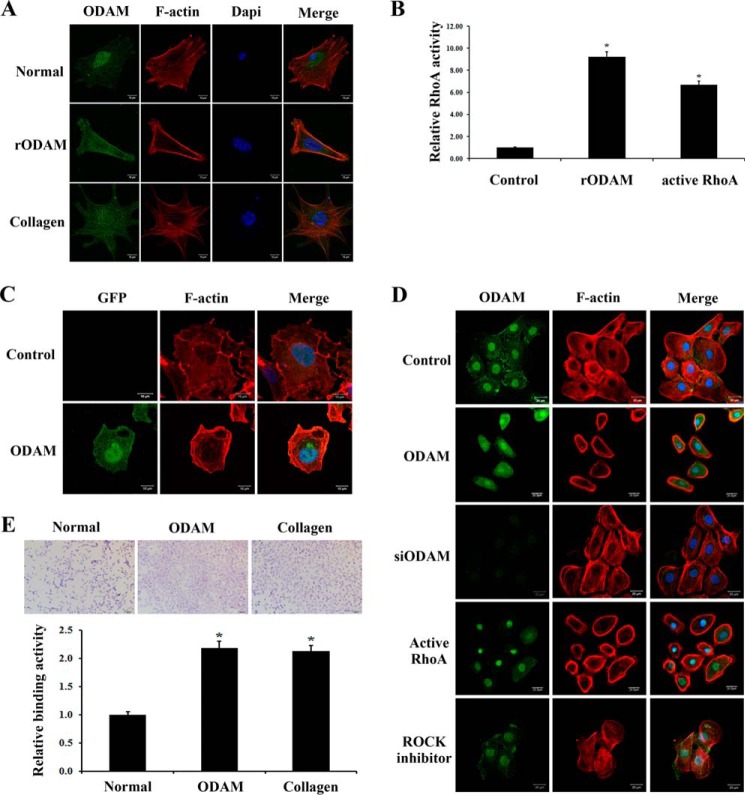
**ODAM induced actin rearrangement in ameloblasts via RhoA signaling.** *A*, cells were cultured on rODAM- or collagen-coated slides for 24 h. Fixed cells were treated with rhodamine-phalloidin to examine actin filament rearrangement using confocal laser microscopy (*red*). ODAM localization was investigated by immunofluorescence. *Scale bars* = 10 μm. *B*, ALCs were treated with rODAM or transfected with active *RhoA* constructs. Equal amounts of cell lysates were used for the G-LISA RhoA activation assay. *C*, ALCs were transfected with *ODAM*, and rhodamine-phalloidin was used to examine the arrangement of actin filaments (*red*). *Scale bars* = 10 μm. *D*, *ODAM*, *ODAM* siRNA, or active *RhoA* constructs were transfected into ALCs. ALCs were treated with ROCK inhibitor (Y-27632). Rhodamine-phalloidin was used to examine the arrangement of actin filaments (*red*). ODAM localization was investigated by immunofluorescence. *Scale bars* = 20 μm. *E*, adhesion of ALCs to ODAM- or collagen-coated slides. Binding values are on the basis of the absorbance of adherent cells. Data are presented as mean ± S.D. of triplicate experiments. *, *p* < 0.05 compared with the control.

As the cell reorganizes from a short epithelial cell to a secretory ameloblast, to a shorter cell able to alter its apical surface, and, finally, to a protective ameloblast, the actin cytoskeleton must reorganize continuously (27, 28). To investigate whether ODAM could affect F-actin distribution, we cultured ALCs for 24 h on rODAM- or collagen-coated slides and examined ODAM and F-actin expression. Cells cultured on rODAM protein showed a greater density of F-actin filaments at the cell periphery compared with cells cultured on collagen (Fig. 5*A*). To confirm the effects of ODAM on RhoA activation, we examined the activation levels of RhoA using a G-LISA RhoA activation assay after rODAM treatment. RhoA signaling was powerful in rODAM-treated and active *RhoA*-expressing ALCs compared with the control (Fig. 5*B*).

Next, we evaluated subcellular alterations in F-actin after exogenous *ODAM* expression in ameloblasts. Confocal microscopy showed specific localization of GFP-tagged ODAM in the nucleus and cytoplasm of ALCs and F-actin accumulated at the cell edge compared with the control (Fig. 5*C*). To determine which functional domain of ODAM is responsible for actin rearrangement and cell shape, several *ODAM* deletion mutants were generated, and cells were examined using immunofluorescence analyses. ODAM and RhoA overexpression resulted in a greater density of F-actin filaments at the cell periphery compared with cells transfected with the ODAM siRNA construct or treated with ROCK inhibitor (Y-27632) (Fig. 5*D*). We also investigated whether ODAM could affect the adhesion of ameloblasts to the substrate by adhesion assay. ODAM- and collagen-coated ALCs exhibited significantly increased cell adhesion compared with control (Fig. 5*E*). These results suggest that ODAM-mediated RhoA signaling resulted in actin filament rearrangement at the cell periphery of ameloblasts with promotion of cell adhesion.

##### Integrin-mediated ODAM Expression Induced RhoA Signaling

**FIGURE 6.**
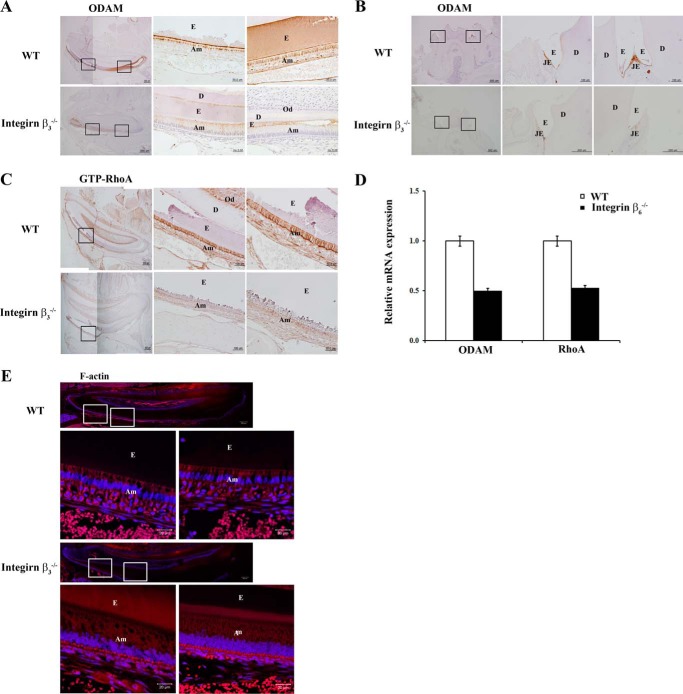
**Integrin β_3_ depletion diminishes ODAM, ARHGEF5, and RhoA expression in ameloblasts and JE.** Tooth sections from *integrin* β*_3_*^−/−^ mice were evaluated. *A–C*, ODAM (*A* and *B*) and GTP-RhoA (*C*) protein expression was detected in ameloblasts and JE from WT and *integrin* β*_3_*^−/−^ mice aged 9 weeks by immunohistochemistry (*n* = 3/group). *Scale bars* = 500, 200, 100, or 50 μm. *E*, enamel; *Am*, ameloblast; *D*, dentin; *Od*, odontoblast. *D*, *ODAM* and *RhoA* mRNA expression was analyzed from gene expression dataset GSE2255 deposited in the GEO (*n* = 5). *E*, F-actin expression was detected by immunofluorescence in teeth and JE of WT and *integrin* β*_3_*^−/−^ mice aged 9 weeks. *Scale bars* = 20 μm.

Integrin β_3_ is required for proper growth of the cervical loop, promotion of the proliferation of preameloblastic cells, and iron transportation during enamel formation (29, 30). *Integrin* β*_3_*^−/−^ mice exhibited shorter lower incisors; similarly, *integrin* β*_6_*^−/−^ mice have severe attrition and an abnormal enamel surface (30, 31). To examine whether integrin could affect ODAM and RhoA expression in ameloblasts, we immunohistochemically analyzed *integrin* β*_3_*^−/−^mice. In the incisor, ODAM was strongly expressed in maturation-stage ameloblasts of WT mice, but its expression was reduced in *integrin* β*_3_*^−/−^ mice (Fig. 6*A*). Interestingly, WT JE was strongly immunolabeled with the ODAM antibody but was hardly expressed in JE of *integrin* β*_3_*^−/−^ mice (Fig. 6*B*). To investigate whether integrin β_3_disruption also affects the expression of GTP-RhoA in ameloblasts, we performed immunostaining in the molar tooth of WT or *integrin* β*_3_*^−/−^ mice. In *integrin* β*_3_*^−/−^ mice, ameloblasts showed little immunoreactivity with the GTP-RhoA antibody. In contrast, WT ameloblasts showed strong GTP-RhoA expression (Fig. 6*C*).

Integrin α_v_β_6_ is expressed in ameloblasts, and it plays a crucial role in regulating amelogenin deposition and/or turnover and subsequent enamel biomineralization (31). We analyzed GEO data using alveolar macrophages in *integrin* β*_6_*^−/−^ mice. ODAM and RhoA expression were decreased significantly in alveolar macrophages of *integrin* β*_6_*^−/−^ mice compared with WT mice (Fig. 6*D*).

When RhoA is activated, ROCK increases actin stress-fiber formation (32). We examined the effects of integrin β_3_ disruption on actin arrangement in ameloblasts from 9-week-old WT and *integrin* β*_3_*^−/−^ mice incisors. In WT incisors, F-actin was distributed throughout the cytoplasm of ameloblasts and was concentrated at both the apical and basal ends. However, in *integrin* β*_3_*^−/−^ incisors, F-actin was weakly and diffusely detected in ameloblasts without polarity (Fig. 6*E*). Overall, these data indicate that integrin β_3_ and β_6_ expression is important for cytoskeleton reorganization via integrin-ODAM-ARHGEF5-RhoA signaling in ameloblasts and JE.

##### Fibronectin and Laminin Activated Integrin-mediated ODAM Signaling

**FIGURE 7.**
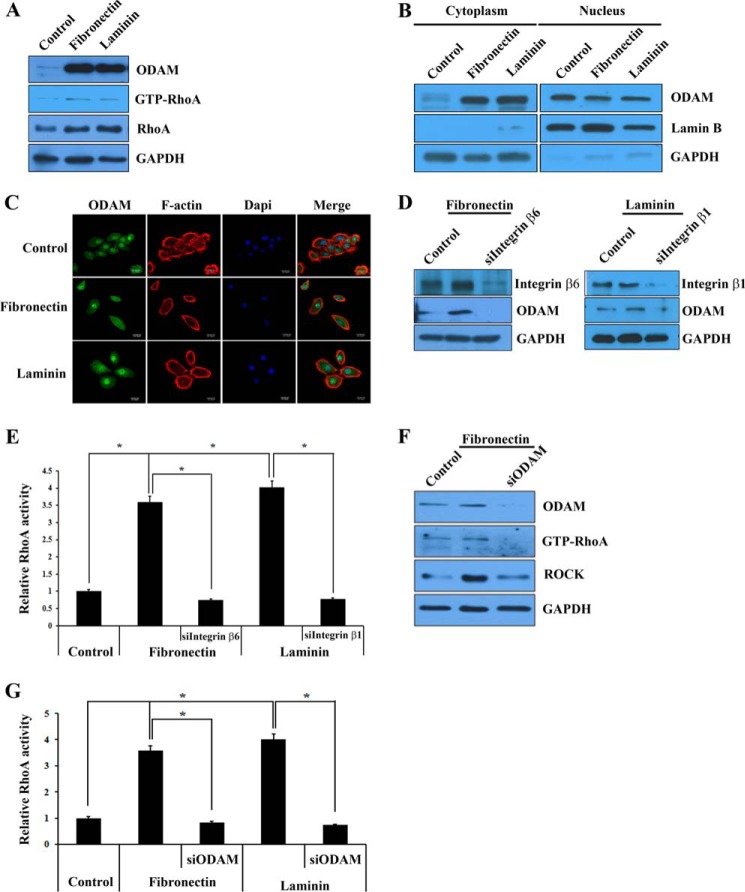
**Fibronectin and laminin activated integrin-ODAM signaling.** *A*, ODAM and GTP-RhoA protein expression were evaluated in ameloblastic HAT7 cells after fibronectin or laminin treatment by Western blot. *B*, effect of fibronectin and laminin on ODAM expression and localization in HAT7 cells by Western blot. *C*, immunofluorescence staining of ODAM (*green*) and F-actin (*red*) in HAT7 cells after fibronectin or laminin treatment. *Scale bars* = 20 μm. *D*, ODAM expression levels were evaluated in ALCs by Western blot transfected with *integrin* β*_6_* or β*_1_* siRNA for 48 h after fibronectin or laminin treatment. *E*, ALCs were treated with fibronectin or laminin and then transfected with *integrin* β*_6_* or β*_1_* siRNA. Equal amounts of cell lysates were used for the G-LISA RhoA activation assay. *F*, ODAM, GTP-RhoA, and ROCK expression levels were evaluated in ALCs by Western blot transfected with *ODAM* siRNA for 48 h after fibronectin treatment. *G*, ALCs were treated with fibronectin or laminin and then transfected with *ODAM* siRNA. Equal amounts of cell lysates were used for the G-LISA RhoA activation assay. *, values significantly different from control (*p* < 0.05).

Fibronectin and laminin, which are components of the basement membrane, participate in the proliferation, differentiation, and attachment of preameloblasts and JE (33–36). In addition, integrins associated with the cytoskeletal proteins fibronectin and laminin regulate cellular processes such as cell adhesion and differentiation (37). To examine whether fibronectin and laminin could induce ODAM-RhoA signaling via integrin, we investigated ODAM and RhoA expression in ameloblastic HAT7 cells after fibronectin or laminin treatment by Western blot. The expression levels of ODAM, RhoA, and active RhoA were increased in HAT7 cells treated with fibronectin and laminin compared with the control (Fig. 7*A*). To investigate whether fibronectin or laminin could induce the localization and expression of ODAM and F-actin, we cultured HAT7 cells with fibronectin or laminin and then evaluated ODAM and F-actin expression by Western blot and immunofluorescence. Cytoplasmic ODAM expression in HAT7 cells was increased significantly by fibronectin and laminin treatment, but nuclear ODAM was decreased slightly (Fig. 7*B*). Cytoplasmic ODAM expression was more intensive in fibronectin- or laminin-treated HAT7 cells than in control cells. Fibronectin- or laminin-treated cells showed a nearly complete disappearance of central actin stress fibers with a transition to circumferential actin cables (Fig. 7*C*).

The lack of integrin β_6_ and β_1_could contribute to the periodontal phenotype (17). We examined the effects of integrin β_6_ and β_1_ disruption in JE. In ameloblasts, increased ODAM expression by fibronectin or laminin was reversible by the addition of *integrin* β*_6_* or β*_1_* siRNA (Fig. 7*D*). To confirm RhoA activation under these conditions, we investigated RhoA activity in ameloblasts. Surprisingly, RhoA signaling was robust in fibronectin- or laminin-treated ameloblasts, similar to ODAM-expressing cells, but inhibited by siRNA-mediated *integrin* inactivation (Fig. 7*E*). To confirm whether ODAM could be regulated by fibronectin or laminin-integrin signaling, we investigated RhoA activity after fibronectin or laminin treatment and then ODAM siRNA transfection. ODAM, GTP-RhoA, and ROCK expression were increased by fibronectin and were reversible by the addition of *ODAM* siRNA (Fig. 7*F*). In addition, RhoA activity was enhanced by fibronectin or laminin but inhibited by siRNA-mediated *ODAM* inactivation (Fig. 7*G*). These results indicate that fibronectin and laminin activated RhoA signaling, resulting in actin reorganization via integrin-mediated ODAM signaling.

##### ODAM Was Re-expressed in Regenerating JE after Gingivectomy in Vivo or Mechanical Scratch in Vitro

**FIGURE 8.**
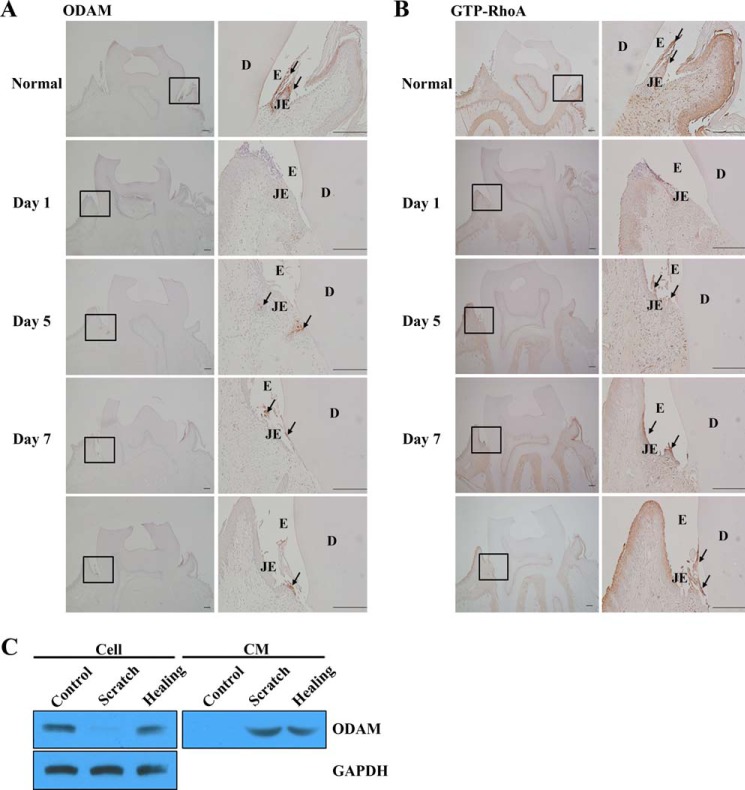
**ODAM was re-expressed in regenerating JE after gingivectomy.** *A* and *B*, ODAM (*A*, *arrows*) and GTP-RhoA (*B*, *arrows*) expression on days 1, 5, and 7 after gingivectomy in regenerating mouse JE by immunohistochemistry (*n* = 2/group). *Scale bars* = 200 μm. *E*, enamel; *D*, dentin. *C*, ODAM expression was evaluated by Western blot analysis in cultured ALCs after scratch wounds.

ODAM expression decreased and subsequently disappeared at damaged JE and epithelial cell rests of Malassez after gingival excision (6, 12, 13). Consistent with these results, during JE regeneration at day 5 after gingivectomy, ODAM was re-expressed in cells at the leading wound edge of the oral epithelium. Immunoreactive cell clusters were also found in the subjacent connective tissue. On day7, ODAM was present in the regenerating JE at the tooth interface (Fig. 8*A*). GTP-RhoA showed a similar expression pattern in regenerating JE after gingivectomy (Fig. 8*B*). In addition, cells damaged by scratching secreted ODAM immediately to the extracellular matrix. However, when the scratch wound healed, ODAM was apparently localized to the cell as well as the extracellular matrix (Fig. 8*C*). These results suggest that intracellular ODAM expression is important for the maintenance of JE attachment to the tooth.

## Discussion

Periodontal diseases are chronic inflammatory processes that affect more than one-third of the adult population and can lead to tooth loss and financial burden (38). Of the utmost importance for maintaining gingival and periodontal health are their defense mechanisms, particularly at the dentoepithelial level. JE, a critical tissue barrier, plays an important role in the formation of epithelial attachment, adhesion of gingiva to the tooth enamel surface, consisting of an internal basal lamina (BL) and hemidesmosomes (1, 3). Peri-implantitis is a key factor responsible for implant failure (39). The attachment of peri-implant epithelium to the titanium surface is similar to the mechanism by which JE cells connect to the natural tooth (40). Peri-implant epithelium is attached to the implant via the internal BL and hemidesmosomes in the lower region of the peri-implant epithelium-implant interface. New findings presented in this paper demonstrate that ODAM functions during JE development and regeneration as well as its functional significance in the initiation and progression of periodontitis and peri-implantitis.

The BL of the JE and peri-implant epithelium is atypical because it constitutively expresses laminin, which contributes to epithelial cell adhesion (41, 42). It is well known that the regenerative JE after gingivectomy is derived from the oral epithelium and that JE maturation is induced by epithelial cell attachment to the tooth surface (43). Immediately after gingivectomy, laminin expression transiently disappeared in the residual tissues (44, 45). However, when the newly formed JE had attached to the enamel surface, lamininγ2 expression was apparent at the internal BL close to the cementoenamel junction, whereas its expression in connective tissue was reduced (46). In this study, laminin activated RhoA signaling, resulting in actin reorganization via integrin-mediated ODAM signaling. After gingivectomy, ODAM expression transiently disappeared but re-expressed upon JE regeneration. Taken together, we suggest that laminin mediates the attachment of JE cells to the internal BL in normal dentogingival junctions and the implant-tooth interface but not in those of inflammatory conditions such as periodontitis and peri-implantitis.

Fibronectin is important for cell adhesion, migration, and differentiation and functions during wound healing by attracting macrophages and other immune cells to the injured area (47). In tooth morphogenesis, fibronectin is synthesized in the dental papilla (48). Fibronectin is associated with the basement membrane separating differentiating ameloblasts and odontoblasts, and further data indicate that this protein is predominantly associated with the filaments of its lamina fibroreticularis (49). In addition, fibronectin is found in the extracellular matrix of periodontium, cartilage, plasma, fibroblasts, and epithelial and endothelial cells and plays a fundamental role in the early stages of healing, promoting cellular migration and tissue regeneration after periodontal treatment (50, 51). Compared with specific laminin expression in internal BL, fibronectin was constitutively expressed in the external BL adjacent to connective tissue (52, 53). In this study, fibronectin activated integrin-ODAM-ARHGEF5-mediated RhoA signaling, which resulted in cytoskeleton reorganization. These results suggest that fibronectin mediates the attachment of JE cells to the external BL in direct contact with the subepithelial connective tissue.

A significant increase in fibronectin and laminin and vitronectin expression was found in human periodontal ligament from teeth treated with orthodontic force for 3 weeks. This result suggests that overexpression of fibronectin and laminin caused ODAM re-expression in regenerating JE after orthodontic tooth movement (54). Apical migration of JE often occurs in association with periodontal inflammation. Laminin was not detected at the migrating tip of JE (52). However, fibronectin has been demonstrated at the migrating tip of epithelial cells in inflammatory periodontium. Therefore, it has been suggested that fibronectin in the subepithelial connective tissue at the apical tip of the migrating epithelium could act as the trigger of cellular migration (53). In this study, in inflammatory periodontium, ODAM disappeared in pathologic pocket epithelium but was detected in GCF. These results suggest that fibronectin in the subepithelial connective tissue induced integrin-mediated ODAM production from migrating epithelial cells. However, although migrating epithelial cells secrete ODAM, the protein was not detected in epithelial cells but in GCF because migrating epithelial cells cannot attach properly to the tooth surface. In addition, the ODAM protein was expressed in GCF from periodontitis and peri-implantitis patients and correlated with probing depth in periodontitis patients. Therefore, we propose that ODAM in GCF could be used as a protein biomarker for periodontitis and peri-implantitis diagnosis.

Integrins play key roles in tooth development because several integrins, including α_6_, α_v_, β_1_, β_3_, β_4_, β_5_, and β_6_ integrin subunits, are expressed in the dental epithelium (55, 56). Integrin α_v_β_6_ is part of the attachment apparatus in the JE that mediates adhesion of the gingival soft tissue to laminin-332 at the enamel interphase of the tooth (15). In this study, ODAM was not expressed in JE of *integrin* β*_3_*^−/−^ mice. There was significantly reduced expression of ARHGEF5, RhoA, and activated RhoA in *integrin* β*_3_*^−/−^ and β*_6_*^−/−^ mice. Our results suggest that integrin α_v_β_3_ and α_v_β_6_ are targets of the ODAM-ARHGEF5-RhoA signaling pathway and play a significant role in tooth-cell adhesion and actin rearrangement during amelogenesis and JE formation.

In summary, we provided experimental evidence for the developmental mechanism of oral epithelial cells such as ameloblasts and JE that attach to the tooth, the mechanism of new attachment occurring after periodontal surgery, and the formation of peri-implant tissue healing in the clinic. Identifying the precise role of ODAM expression in regenerating JE should help clinicians to provide better periodontal care for patients.

## Footnotes

2 The abbreviations used are:

JE: junctional epithelium
ODAM: odontogenic ameloblast-associated protein
GEF: guanine nucleotide exchange factor
GCF: gingival crevicular fluid
PG: *Porphyromonas gingivalis*
DSS: dextran sulfate sodium
ALC: ameloblast lineage cell
rODAM: recombinant odontogenic ameloblast-associated protein
IP: immunoprecipitation
BL: basal lamina
ROCK: Rho-associated kinase.
